# Histopathology Images‐Based Deep Learning Prediction of Histological Types in Endometrial Cancer

**DOI:** 10.1002/cam4.71509

**Published:** 2025-12-30

**Authors:** Lingmei Li, Pengbo Wang, Changyu Geng, Jingyi Wang, Lu Cao, Yanan Gao, Dandan Chen, Ge Qiao, Shi Zhang, Ningrui Feng, Ming Liu, Xiaofeng Li, Yaomei Ma, Su Zhang, Huiting Xiao, Zhongmin Jiang, Xiaozhi Liu, Wenjuan Ma, Lisha Qi

**Affiliations:** ^1^ Department of Pathology Tianjin Medical University Cancer Institute & Hospital, National Clinical Research Center for Cancer, Tianjin's Clinical Research Center for Cancer, Key Laboratory of Cancer Prevention and Therapy Tianjin Tianjin China; ^2^ Department of Breast Imaging Tianjin Medical University Cancer Institute & Hospital, National Clinical Research Center for Cancer, Tianjin's Clinical Research Center for Cancer, Key Laboratory of Cancer Prevention and Therapy Tianjin Tianjin China; ^3^ Department of Molecular Imaging and Nuclear Medicine Tianjin Medical University Cancer Institute & Hospital, National Clinical Research Center for Cancer, Tianjin's Clinical Research Center for Cancer, Key Laboratory of Cancer Prevention and Therapy Tianjin Tianjin China; ^4^ Department of Gynecology Oncology Tianjin Medical University Cancer Institute & Hospital, National Clinical Research Center for Cancer, Tianjin's Clinical Research Center for Cancer, Key Laboratory of Cancer Prevention and Therapy Tianjin China; ^5^ Department of Pathology Tianjin Fifth Central Hospital Tianjin Tianjin China; ^6^ Tianjin Key Laboratory of Epigenetic for Organ Development of Preterm Infants, Tianjin Fifth Central Hospital Tianjin China; ^7^ Department of Pathology, Hotan District People's Hospital Hotan Xinjiang Uygur China

**Keywords:** 2023 International Federation of Gynecology and Obstetrics staging, deep learning, endometrial cancer, histological types

## Abstract

**Background:**

According to the new 2023 International Federation of Gynecology and Obstetrics staging system for endometrial cancer (EC), EC is classified into aggressive and nonaggressive histological types. Accurate diagnosis of the histological type of EC is crucial for optimizing treatment strategies and predicting patient outcomes.

**Objectives:**

To develop and validate a deep convolutional neural network for predicting nonaggressive versus aggressive histological types from hematoxylin and eosin (H&E)‐stained images of EC specimens.

**Methods:**

A deep convolutional neural network named EC‐AI^HIS^ was developed to predict the nonaggressive or aggressive histological type from 1187 EC specimens. Its generalizability and clinical utility were assessed across multiple cohorts and benchmarked against pathological diagnoses. Furthermore, correlations between the model's predictions and molecular subtypes of EC were examined.

**Results:**

EC‐AI^HIS^ achieved an AUC of 0.911 (sensitivity 82%, specificity 83%). In fivefold cross‐validation, AUCs ranged from 0.865 to 0.909. External validation yielded an AUC of 0.859 (sensitivity 75%, specificity 83%). EC‐AI^HIS^ maintained robustness on images from different scanners and of suboptimal quality. In clinical simulation settings, it showed higher sensitivity than pathologists and improved junior pathologists’ diagnostic accuracy. EC‐AI^HIS^ scores were associated with molecular subtypes of EC and showed potential prognostic utility in the p53abn subtype.

**Conclusions:**

EC‐AI^HIS^ is an effective tool that can assist pathologists in classifying EC histological types.

## 1 Introduction

Endometrial cancer (EC) is one of the most prevalent gynecologic malignancies [1, 2, 3]. In 2023, the International Federation of Gynecology and Obstetrics (FIGO) presented a revised staging system for EC, 14 years after 2009 [4]. EC was divided into nonaggressive and aggressive histotypes [5]. Nonaggressive EC is mainly treated with total hysterectomy, while aggressive EC requires more complex surgery and adjuvant treatment [6, 7]. In addition, the new staging system, combined with molecular typing, helps to predict prognosis and guide treatment [8, 9].

The accurate determination of histological type is the prerequisite for correct staging. Although the different EC histological types have different natural histories, precursor lesions, and show characteristic microscopic appearances, many morphologic variations of EC may pose diagnostic challenges for pathologists [10, 11, 12, 13, 14]. Even for experienced gynecologic pathologists, it is not easy to accurately and consistently sub‐classify EC based on morphological features alone, especially for the distinction between low‐grade endometrial endometrioid carcinoma (EEC) and its high‐grade mimics [15, 16, 17].

Over the last decade, the application of artificial intelligence (AI), particularly deep learning (DL), in medicine has increased rapidly. In oncology, the use of DL for diagnostic evaluation and clinical decision‐making mainly depends on the recognition of medical images, such as radiographic and pathologic images. For EC, recent studies have reported that DL has tremendous potential to judge the nature of the lesion, the molecular classification, and the risk factors of EC [18, 19, 20, 21]. DL can also be used to predict tumor recurrence by combining the assessment of medical images, such as colposcopy, hysteroscopy, computed tomography, magnetic resonance images, and histopathological images, with the multi‐omics analysis of clinical data [22, 23, 24, 25]. Nevertheless, research employing DL for the differentiation of nonaggressive versus aggressive histological subtypes in EC remains scarce.

In this study, we developed a DL model, EC‐AI^HIS^, for the prediction of the histotype of EC based on annotated H&E‐stained images and tested its generalizability from multiple independent cohorts. An internal dataset, an external dataset, a dataset with images captured from another scanner, and a dataset with poor‐quality images were used to test the generalizability of EC‐AI^HIS^. To evaluate the practicability of EC‐AI^HIS^ in clinical application, we investigated its performance in a dataset from preoperative endometrial sampling, a dataset with images all showing adenoid structure morphology, and compared its performance with pathologists. Besides, we observed the association between EC‐AI^HIS^ and the molecular subtypes of EC.

## 2 Material and Methods

### 2.1 Study Design and Patient Cohorts

**FIGURE 1:**
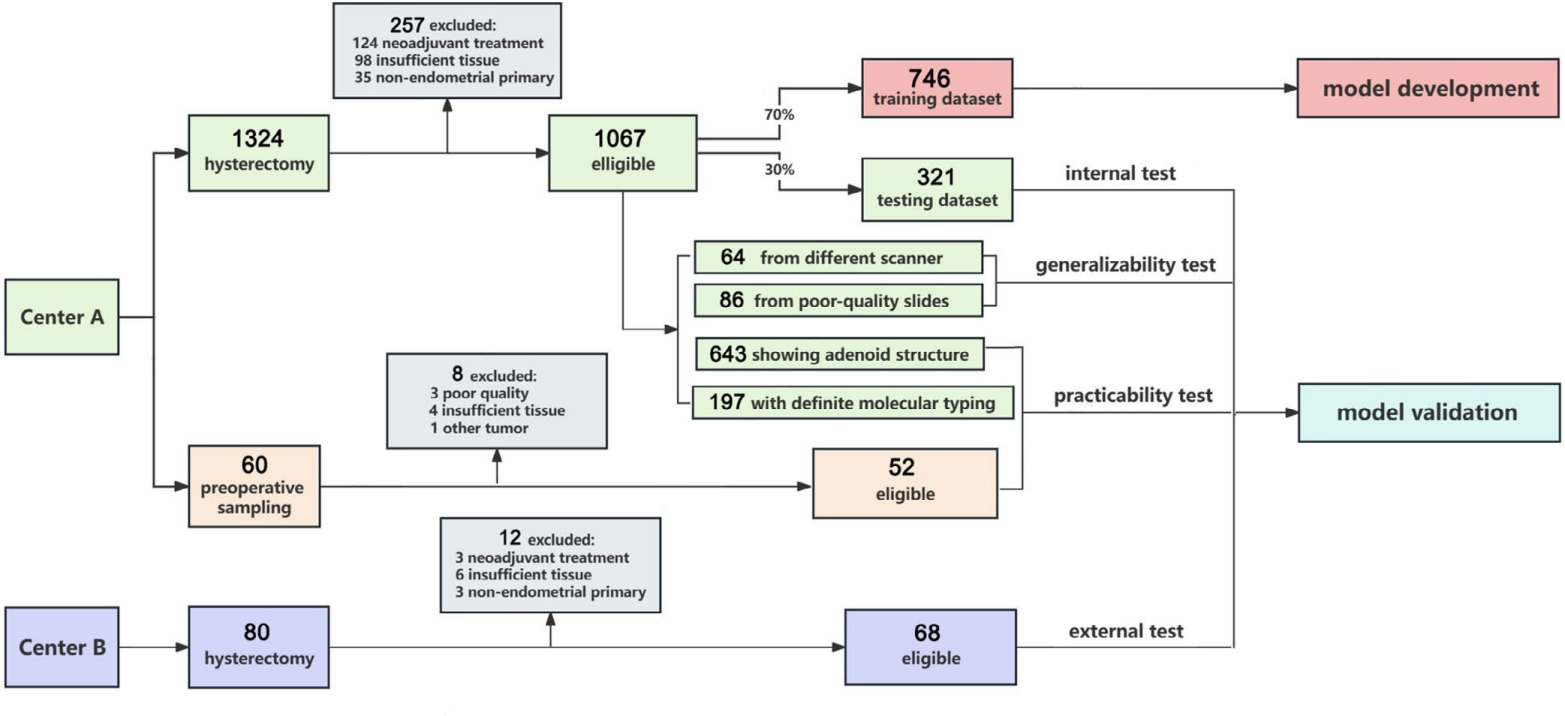
Study design for developing and testing EC‐AI^HIS^ to predict aggressive or nonaggressive histotype of endometrial carcinoma.

A total of 1464 patients with EC were enrolled in this study: 1384 patients from Tianjin Medical University Cancer Institute & Hospital (center A), spanning the period from January 2011 to October 2023, and 80 patients from Tianjin Fifth Central Hospital (center B) from January 2017 to December 2020. Detailed exclusion criteria are presented in Figure 1. The clinic‐pathological data were obtained from hospital records or pathological reports. From Center A, 1067 eligible surgically resected and pathologically confirmed EC specimens were randomly divided into a training set and an internal test set in a 7:3 ratio (746 vs. 321 cases) using a computer‐generated random number sequence. The external test set comprised 68 eligible specimens from patients treated at center B. To test the generalizability of EC‐AI^HIS^, we assembled two additional datasets: one consisting of 64 whole‐slide images obtained using a different scanner model (Motic Slice Scanner, Panthera, Beijing; 50× magnification, 0.2 μm/pixel resolution) compared to the primary scanner (PRECICE Digital, Unina, Beijing), and another containing 86 images of poor‐quality slides (e.g., with knife marks, folding, poor fixation, or suboptimal staining). To test the clinical practicability of EC‐AI^HIS^, a dataset including 643 images showing adenoid‐predominant structure and a dataset including 52 images from preoperative endometrium sampling of EC patients were tested, and the performance of EC‐AI^HIS^ was compared with that of the pathologists. In addition, the performance of EC‐AI^HIS^ was observed in a dataset including 197 images from EC specimens with definite molecular typing. The molecular classification had been done following the surrogate marker‐based diagnostic algorithm of the World Health Organization (WHO) 2020 [26]. Disease‐free survival (DFS) was calculated from the date of diagnosis to the last follow‐up or the date of disease progression, recurrence, or death. The deadline for follow‐up of patients is May 2024.

### 2.2 Selection of H&E‐Stained Histopathology Images

Archival formalin‐fixed paraffin‐embedded (FFPE) tumor sections were retrieved from the pathological specimen repository of Tianjin Medical University Cancer Institute & Hospital and Tianjin Fifth Central Hospital. All the H&E‐stained slides of each patient were reviewed by two experienced gynecological pathologists (Lisha Qi and Yanan Gao) following the diagnostic criteria of the 2020 WHO classification of endometrial tumors. The cases showing adenoid‐predominant structures and the slides with images of poor quality were labeled. For cases with atypical morphological features, consensus was reached after discussion. Next, 1187 representative tissue slides from 1187 cases were chosen and scanned by PRECICE Digital (Unina, Beijing) at 50× magnification (0.2 μm/pixel). Among them, 64 randomly selected H&E slides were also scanned by another digital slide scanner (Motic Slice scanner, Panthera, Beijing). To reduce scanner‐related color variance, ImageNet channel‐wise standardization was applied exclusively.

### 2.3 Image Preprocessing and Development of EC‐AI^HIS^

**FIGURE 2:**
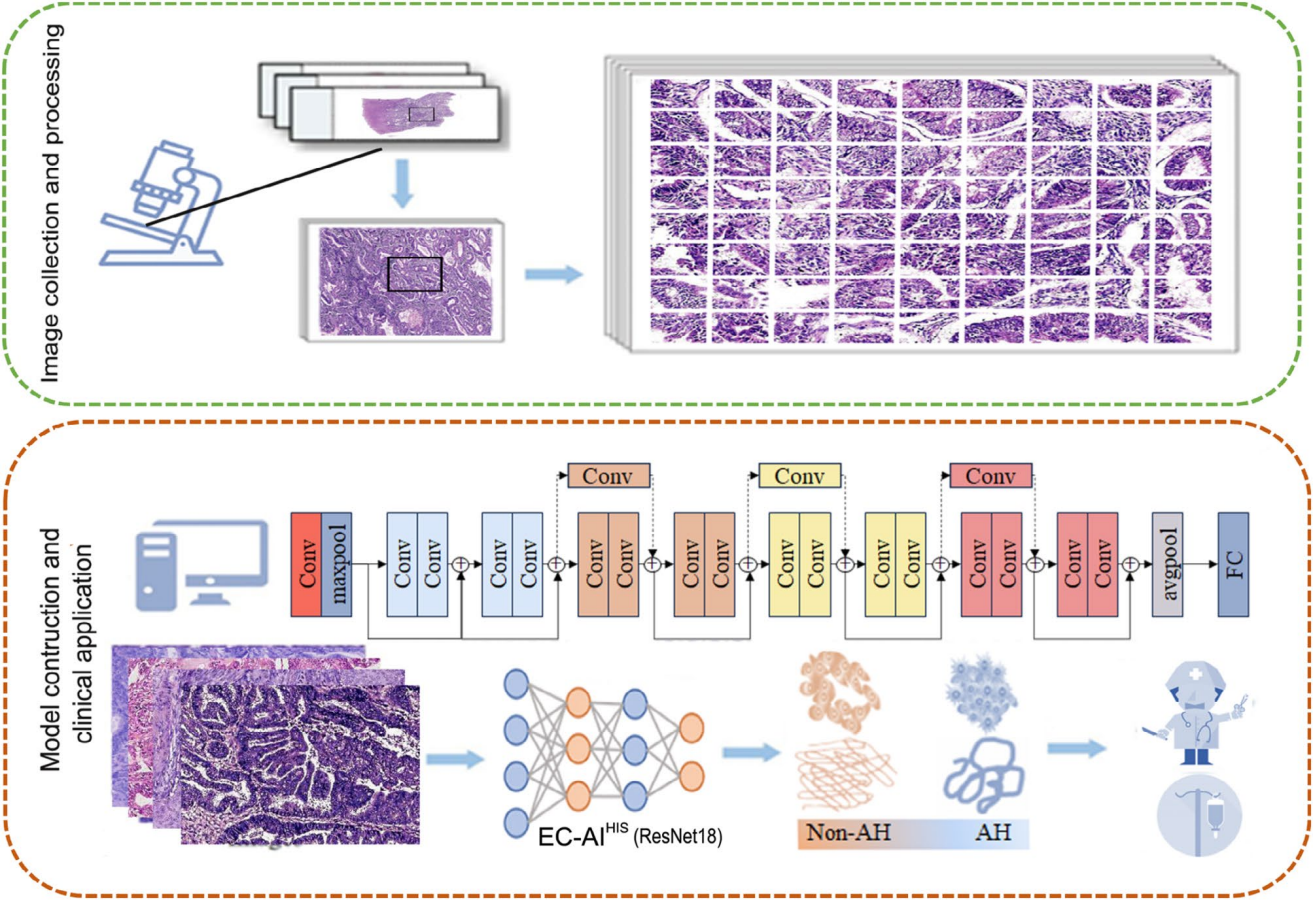
General flowchart demonstrating EC‐AI^HIS^. The H&E‐stained slides were reviewed by pathologists. Representative tissue slides from 1187 EC cases were chosen and scanned at 50× magnification. The representative area was selected for subsequent image preprocessing. The images were normalized and standardized. The EC‐AI^HIS^ model (ResNet18) has all been pretrained on the ImageNet dataset regarding parameter initialization. Fivefold cross‐validation was adopted to verify the effectiveness of the model.

The area with more than 60% tumor purity in each digitized slide was delineated for subsequent image preprocessing. All the annotation areas in the high‐resolution digital images (224 × 224 pixels) were extracted. The images were randomly flipped vertically and horizontally with a probability of 0.5 for each transformation. No additional augmentation was applied. The images were normalized and standardized, where the mean and standard deviation on the (R, G, and B) channels are (0.485, 0.456, and 0.406) and (0.229, 0.224, and 0.225), respectively, which are the mean and standard deviation of the ImageNet dataset. The EC‐AIHIS model is based on the structure of the ResNet18 model, which consists of five modules. The first module is the initial convolutional layer, and the subsequent four modules (Layers 1–4) are composed of multiple residual blocks. Each residual block contains two convolutional layers, and communication between different residual blocks is achieved through residual connections. Finally, a fully connected layer is used to output the results. The EC‐AI^HIS^ model (ResNet18) has all been pretrained on the ImageNet dataset regarding parameter initialization [27]. Regarding network architecture, the output length of the last fully connected layer of the EC‐AI^HIS^ model is adjusted to 2. We used the cross‐entropy function as the model's loss function and the AdamW optimizer for training. The number of training epochs was set to 200. For the learning rate scheduling strategy, we employed the CosineAnnealingLR scheduler, with the maximum learning rate set to 0.00001 and Tmax set to 100. Regarding hyperparameters, the batch size was 32, and the initial learning rate was 0.00001. Fivefold cross‐validation was adopted to verify the effectiveness of the model. Patient‐level randomization was conducted using a fixed numpy random seed (random seed = 42). The general flowchart of the training, testing, and visualization followed the workflow in Figure 2. The random seed, exact data‐split logic, and augmentation pipeline are recorded in the internal experimental log and can be shared in pseudonymized form under appropriate institutional ethical approval.

### 2.4 EC‐AI^HIS^ Versus Pathologists

To compare the performance of EC‐AI^HIS^ with that of pathologists, we randomly selected 197 images from the 643‐case dataset showing adenoid structure and all 52 images from the preoperative endometrium samples for manual interpretation. Four practicing pathologists (two senior gynecologic pathologists with 8 years of experience, two junior pathologists with 3 years of experience) checked all the 249 selected images independently without reference to immunohistochemistry results. To investigate whether EC‐AI^HIS^ could improve the junior pathologists' diagnostic ability, 191 additional images (not overlapping with the 197 and 52 images) were randomly selected from the internal testing sets for the two junior pathologists, with the corresponding prediction results from EC‐AI^HIS^ as reference.

### 2.5 Image Feature Visualization

Attention heat maps were utilized to visualize image features learned by EC‐AI^HIS^. In aggregate, 321 images were used to generate corresponding attention heat maps. Two senior pathologists assessed the attention heat maps and analyzed the correlation of image features to histopathological interpretations.

### 2.6 Statistical Analysis

Data were analyzed using SPSS software (version 15.0). The continuous variables were described with means or medians, and categorical variables were summarized with frequencies or percentages. Univariate analysis was used to evaluate the clinical factors in the training set, and the factors with a *p* < 0.05 were combined with the EC‐AI^HIS^ results, and the prediction efficiency of the XGboost method was compared with EC‐AI^HIS^ alone. The Kaplan–Meier method and log‐rank test were used to analyze the estimated survival. A *p* < 0.05 was considered to be statistically significant. To measure model performance, the AUC, sensitivity, specificity, positive predictive value (PPV), negative predictive value (NPV), accuracy, and F1 score were calculated. The DL model computation and data analysis processes were implemented with Python (3.8.13) and R (version 3.4.0).

## 3 Results

### 3.1 Patient Characteristics and Image Datasets

The clinic‐pathological data, including age, vaginal bleeding, menarche age, menopause status, childbirth history, abortion, family history, histological type, myometrial invasion, the extent of lympho‐vascular space invasion (LVSI), lymph node metastasis, and cervix invasion, are shown in Table 1.

**TABLE 1:** Clinicopathological characteristics of endometrial carcinoma patients.

Characteristics	Cohort in center A (*n* = 1067)	Cohort in center B (*n* = 68)
Age, mean (range)		
≤ 50	285 (26.7)	16 (23.5)
> 50	782 (73.3)	52 (76.5)
Vaginal bleeding		
No	162 (15.2)	12 (17.7)
Yes	905 (84.8)	56 (82.3)
Menarche age		
≤ 14	543 (50.9)	35 (51.5)
> 14	524 (49.1)	33 (48.5)
Menopause status		
No	364 (34.1)	17 (25.0)
Yes	703 (65.9)	51 (75.0)
Childbearing history		
No	62 (5.8)	4 (5.9)
Yes	1005 (94.2)	64 (94.1)
Abortion		
No	428 (40.1)	28 (41.2)
Yes	639 (59.9)	40 (58.8)
Family history		
No	803 (75.3)	52 (76.5)
Yes	264 (24.7)	16 (23.5)
Histological type		
Nonaggressive	750 (70.3)	32 (47.1)
Aggressive	317 (29.7)	36 (52.9)
Myometrial invasion		
< 1/2	892 (83.5)	45 (66.2)
≥ 1/2	156 (14.6)	23 (33.8)
Uncertain	19 (1.8)	0 (0)
The extent of LVSI		
No/focal	962 (90.1)	54 (79.4)
Substantial	105 (9.8)	14 (20.6)
Lymph node metastasis		
No	559 (52.3)	56 (82.3)
Yes	40 (3.7)	7 (10.3)
Uncertain	468 (43.8)	5 (7.4)
Cervix invasion		
No	997 (93.4)	60 (88.2)
Yes	70 (6.6)	8 (11.8)

Abbreviation: LVSI, lympho‐vascular space invasion.

The training dataset consisted of 746 images covering eight tumor subtypes: endometrial endometrioid carcinoma (EEC), endometrial serous carcinoma (ESC), endometrial clear cell carcinoma (ECCC), endometrial mixed carcinoma (EMixC), endometrial carcinosarcoma (ECS), endometrial undifferentiated carcinoma (EUC), endometrial dedifferentiated carcinoma (EDC), and mesonephric‐like adenocarcinoma (MLA). Nonaggressive EC amounted to 70.1% (*n* = 523) of overall cases, among which the cases of grade 2 EEC (40.0%, *n* = 298) were slightly more than grade 1 EEC (30.2%, *n* = 225). The proportion of aggressive EC was 29.9% (*n* = 223), among which ESC and grade 3 EEC comprised the two largest categories (21.4%, *n* = 160), followed by other rare histological types. The histology categories of different testing datasets were broadly in line with those of the training set (Table 2).

**TABLE 2:** Histological subtypes of nonaggressive and aggressive endometrial carcinoma in the datasets.

Category	Sub‐category	Internal dataset (%)	External dataset (%)	Different scanner (%)	Poor quality (%)	Curettage dataset (%)	Total
Nonaggressive histological type		750 (70.3)	29 (42.6)	31 (48.4)	42 (48.8)	38 (73.1)	890
	EEC, Grade 1	354 (47.2)	14 (48.3)	9 (29.0)	22 (52.4)	20 (52.6)	419
	EEC, Grade 2	396 (52.8)	15 (51.7)	22 (71.0)	20 (47.6)	18 (47.4)	471
Aggressive histological type		317 (29.7)	39 (57.4)	33 (51.6)	44 (51.2)	14 (26.9)	447
	EEC, Grade 3	151 (47.6)	7 (17.9)	1 (3.0)	3 (6.8)	2 (14.3)	164
	ESC	107 (33.8)	24 (61.6)	15 (45.6)	35 (79.5)	6 (43.0)	187
	ECCC	23 (7.3)	1 (2.6)	7 (21.2)	1 (2.3)	2 (14.3)	34
	EMixC	18 (5.7)	7 (17.9)	1 (3.0)	0	1 (7.1)	27
	ECS	11 (3.4)	0	6 (18.2)	3 (6.8)	1 (7.1)	21
	EUC	4 (1.3)	0	1 (3.0)	1 (2.3)	1 (7.1)	7
	EDC	2 (0.6)	0	0	1 (2.3)	0	3
	MLA	1 (0.3)	0	2 (6.0)	0	1 (7.1)	4
Total		1067	68	64	86	52	1337

Abbreviations: ECCC, endometrial clear cell carcinoma; ECS, endometrial carcinosarcoma; EDC, endometrial dedifferentiated carcinoma; EEC, endometrial endometrioid carcinoma; EMixC, endometrial mixed carcinoma; ESC, endometrial serous carcinoma; EUC, endometrial undifferentiated carcinoma; MLA: mesonephric‐like adenocarcinoma.

### 3.2 Performance of EC‐AI^HIS^ on Prediction of Aggressive or Nonaggressive Histologic Types of EC

**FIGURE 3:**
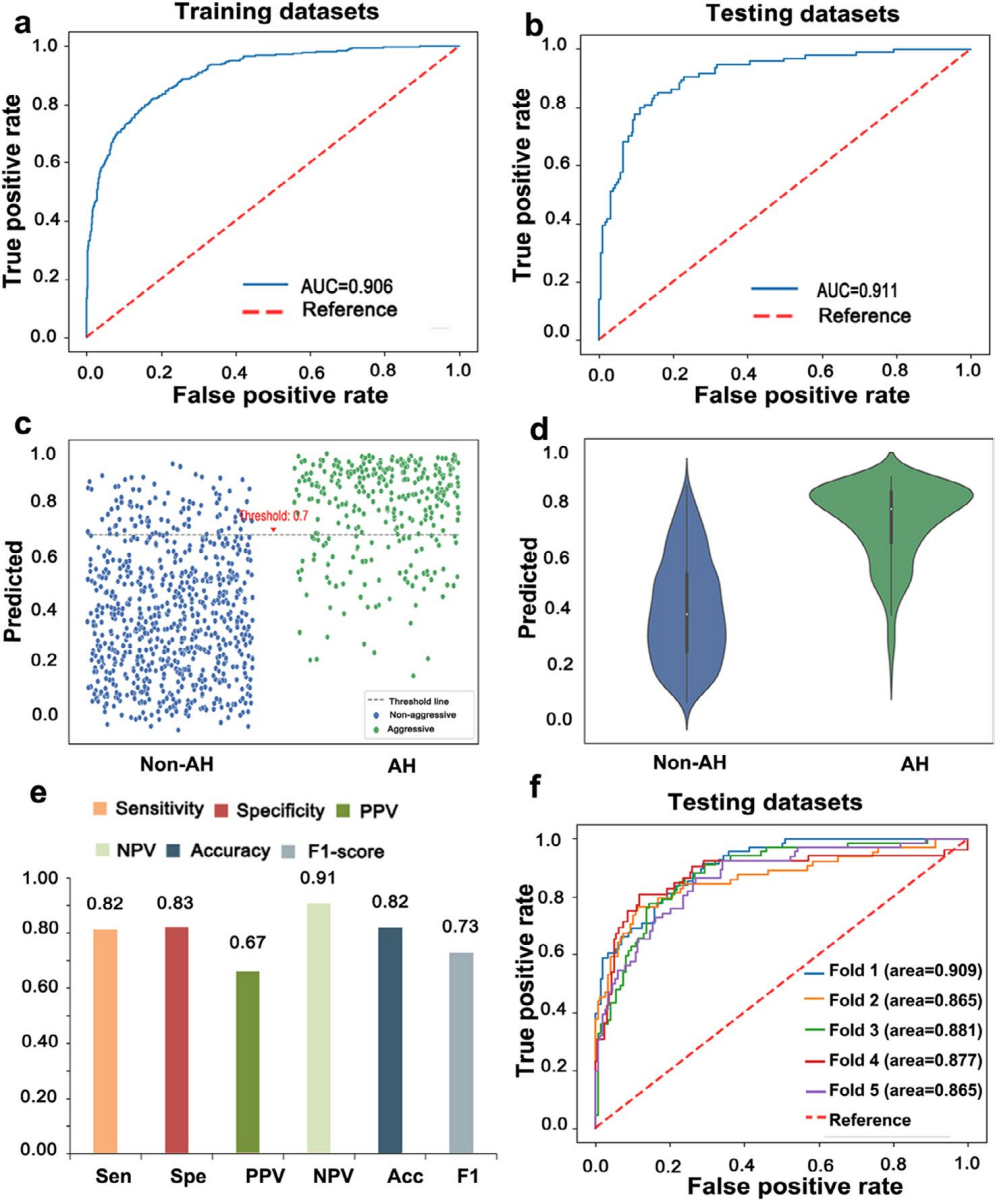
Performance of EC‐AI^HIS^ in the internal dataset. (a) and (b) The AUC of EC‐AI^HIS^ for training (a) and testing (b) datasets in the internal dataset. (c) and (d) The overview of the distribution of predicted values in nonaggressive histological type and aggressive histological type. (e) Sensitivity, specificity, positive predictive value (PPV), negative predictive value (NPV), accuracy, and F1‐score of EC‐AI^HIS^. (f) The fivefold cross‐validation results of a deep learning model with an AUC ranging from 0.865–0.909 in testing datasets.

As shown in Figure 3, EC‐AI^HIS^ attained performance with an average AUC of 0.906 and 0.911 for training datasets and testing datasets, respectively (Figure 3a,b,e). The distribution of predicted values was also generated and referred to evaluate the prediction task. The classification results matched well with the nonaggressive histological types and aggressive histological types (Figure 3c,d). In addition, EC‐AI^HIS^ achieved similar results in the fivefold cross‐validation, showing stable effectiveness in the two‐class classification task. The AUCs were 0.865 and 0.909 for the folds with the lowest and highest AUCs, respectively (Figure 3f).

We attempted to add the clinical information as an additional predictor to improve the accuracy of EC‐AI^HIS^. Age and menopause status significantly differed between the aggressive and nonaggressive groups (Table S1). However, the calibration curve of combined clinical criteria and EC‐AI^HIS^ did not more accurately predict the classification of the histologic types than EC‐AI^HIS^ alone (Figure S1).

**FIGURE 4:**
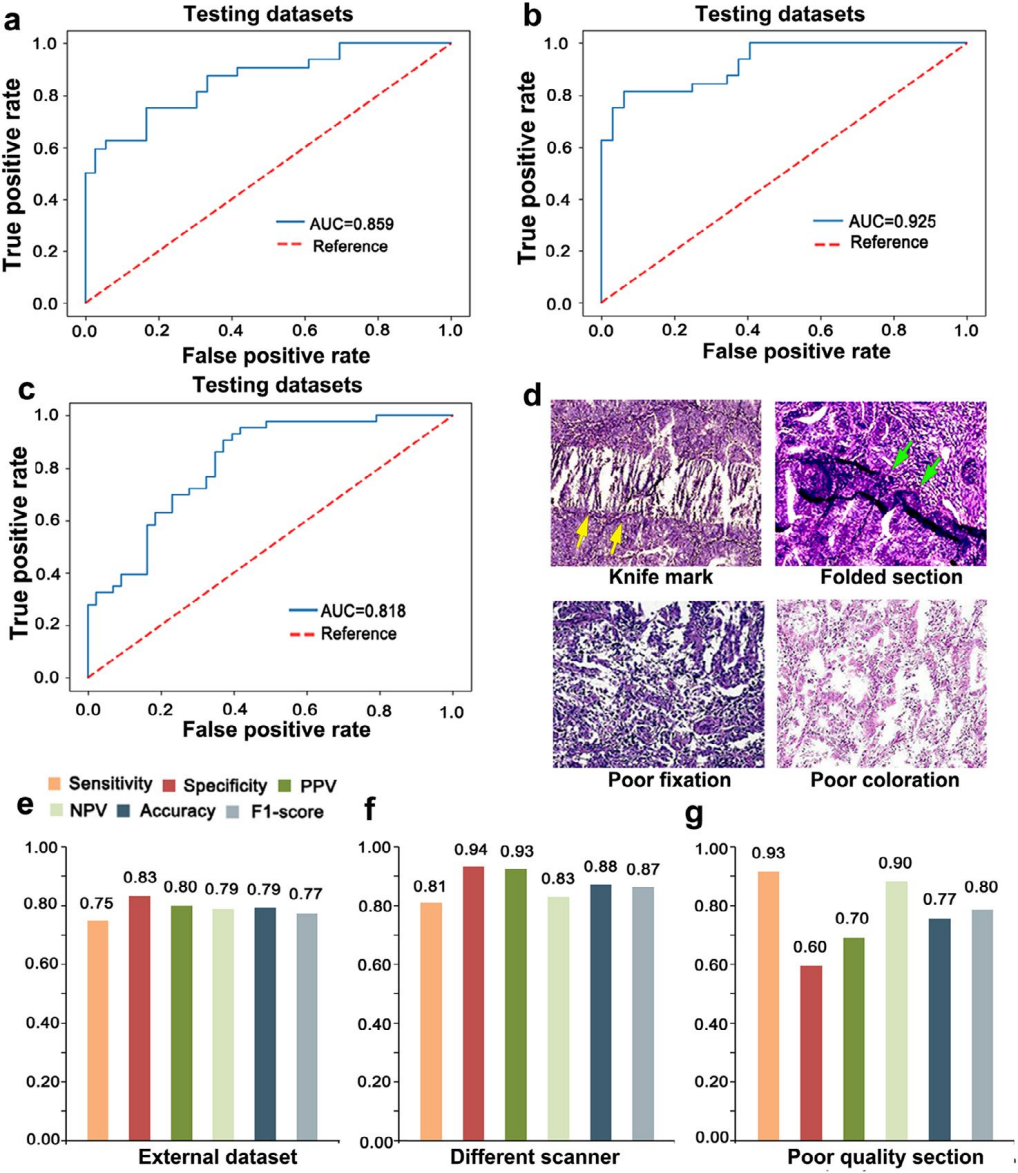
Performance of EC‐AI^HIS^ in the datasets from external center, different scanner, and poor‐quality slides. (a–c) The AUC of EC‐AI^HIS^ on the external dataset (a), another scanner dataset (b), and poor‐quality slides dataset (c). (d) The morphology images with knife marks (marked with yellow arrows), folded sections (marked with green arrows), poor fixation, and color features. (e–g) Sensitivity, specificity, positive predictive value (PPV), negative predictive value (NPV), accuracy, and F1‐score of EC‐AI^HIS^ in the external dataset (e), different scanners dataset (f), and poor‐quality slides dataset (g).

On the external testing set, EC‐AI^HIS^ achieved an AUC of 0.859 with a 75% sensitivity and 83% specificity (Figure 4a,e). Moreover, PPV and NPV values reached 80% and 79%, respectively. In addition, in the course of daily pathological diagnosis, it is inevitable to encounter situations of analyzing the images captured by different scanners or images from poor‐quality slides caused by various reasons, including knife marks, folded sections, poor fixation, and poor coloration, as shown in Figure 4d. To further verify the generalization and reliability of EC‐AI^HIS^, we enrolled 64 images captured by another scanner, different from the one used in the training set, and 86 images from poor‐quality slides. EC‐AI^HIS^ achieved satisfactory performance in both datasets, with an AUC of 0.925 and 0.818, accuracy of 88% and 77%, respectively (Figure 4b,c,f,g). These classification results match well with the overall performance of EC‐AI^HIS^ in the training set and the fivefold cross‐validation process.

### 3.3 Performance of EC‐AI^HIS^ Versus Pathologists

**FIGURE 5:**
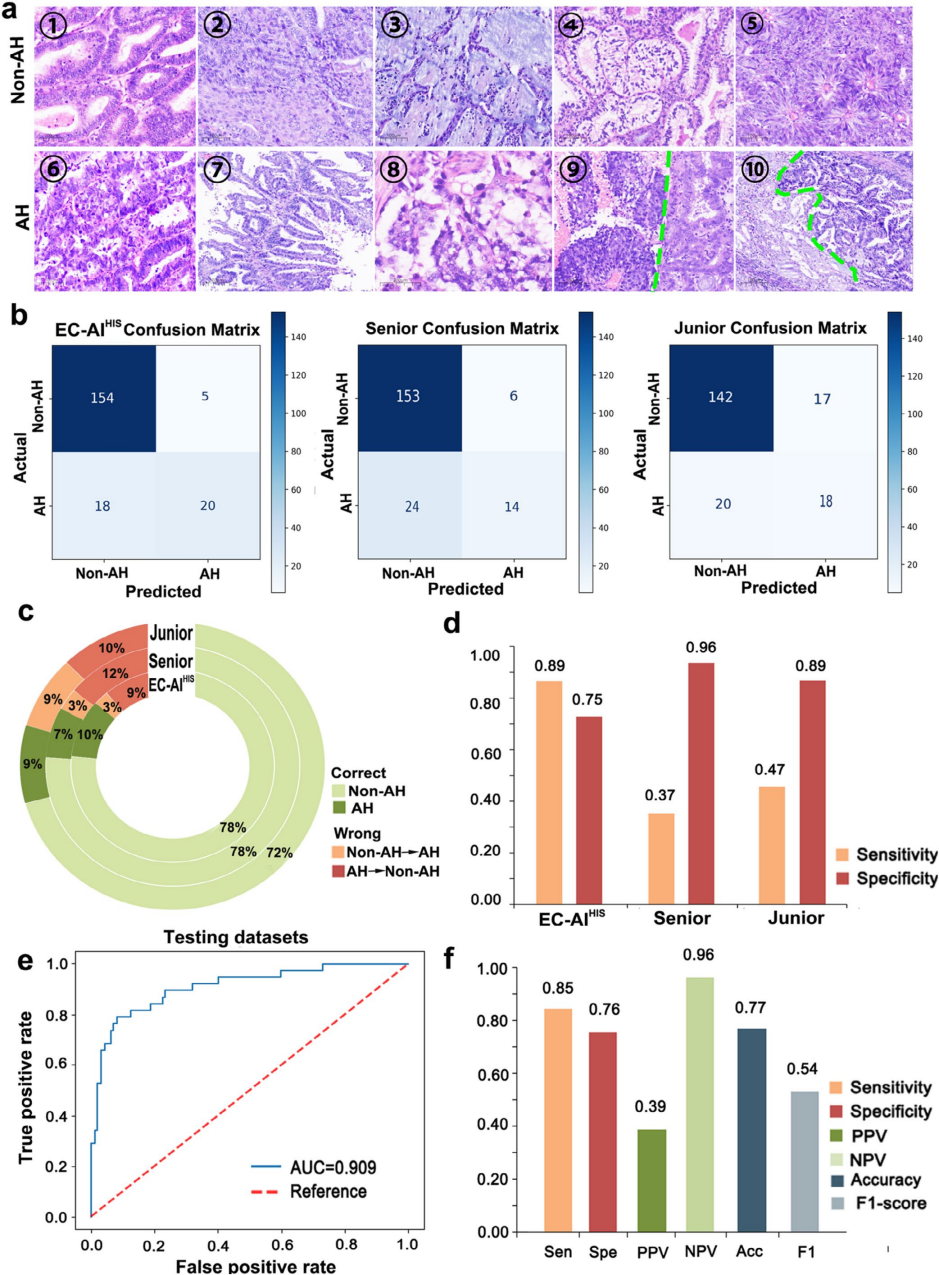
Performance of EC‐AI^HIS^ and comparison with pathologists in the dataset showing adenoid structure (a) The representative histological images of different EC histological subtypes showing adenoid structure. ① low‐grade endometrioid carcinoma; ② endometrioid carcinoma with squamous differentiation; ③ endometrioid carcinoma with mucinous pattern; ④ endometrioid carcinoma with secretory patterns; ⑤ endometrioid carcinoma with sertoliform pattern; ⑥ endometrial serous carcinoma; ⑦ endometrial serous carcinoma with papillary structure; ⑧ endometrial clear cell carcinoma; ⑨ endometrial dedifferentiated carcinoma (Undifferentiated carcinoma is on the left of the green dotted line, and endometrioid carcinoma is on the right); ⑩ endometrial mixed carcinoma (Serous carcinoma is on the left of the green dotted line, and endometrioid carcinoma is on the right).(b–d): The comparison between EC‐AI^HIS^ and pathologists. (e) The AUC of EC‐AI^HIS^ in the dataset showing adenoid structure. (f) Sensitivity, specificity, positive predictive value (PPV), negative predictive value (NPV), accuracy, and F1‐score of EC‐AI^HIS^ in the dataset showing adenoid structure.

**FIGURE 6:**
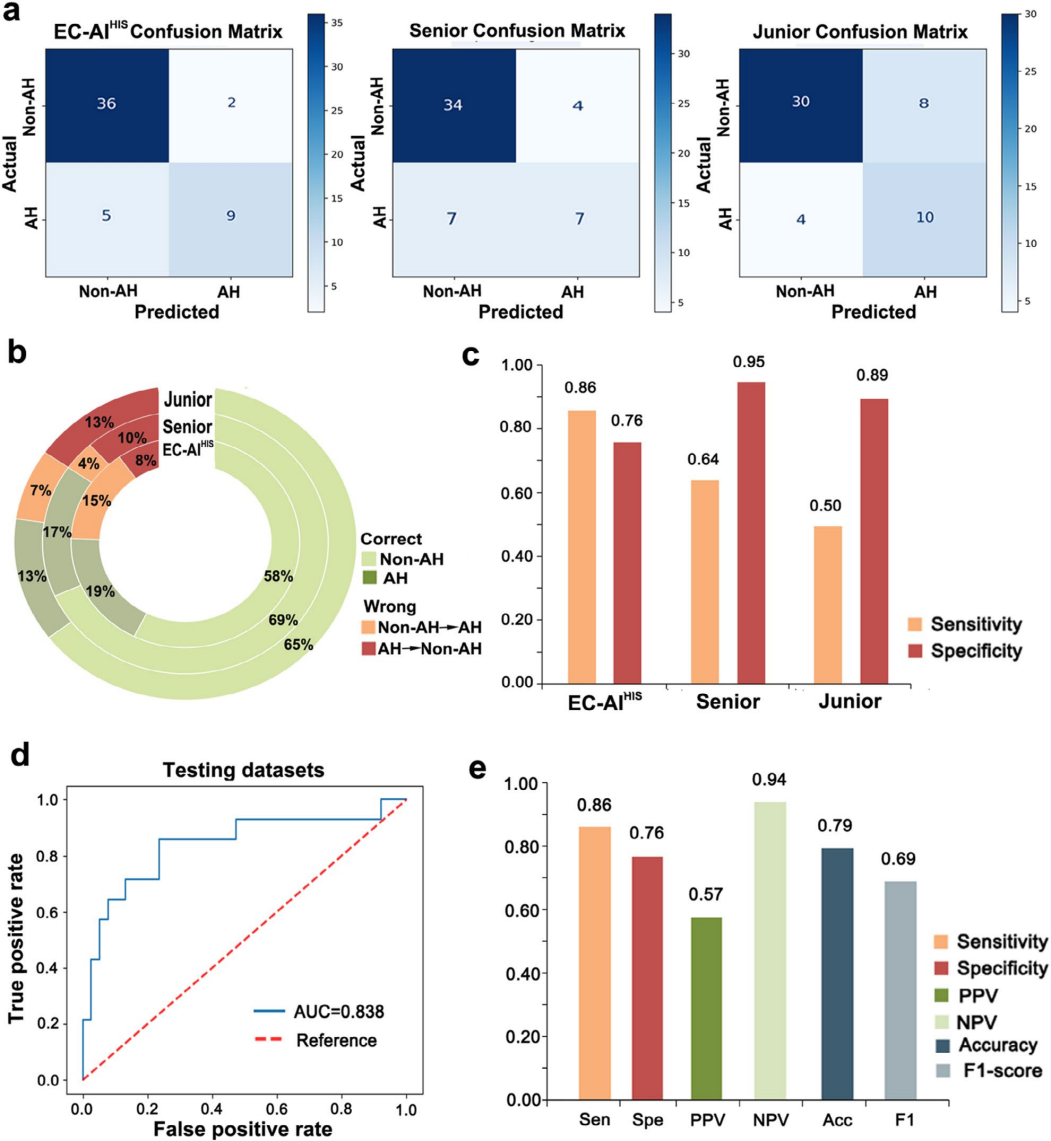
Performance of EC‐AI^HIS^ and comparison with pathologists in the dataset with images from preoperative endometrial biopsy samples. (a–c) The comparison between EC‐AI^HIS^ and pathologists. (d) The AUC of EC‐AI^HIS^ in the dataset with images from preoperative endometrial biopsy samples. (e) Sensitivity, specificity, positive predictive value (PPV), negative predictive value (NPV), accuracy, and F1‐score of EC‐AI^HIS^ in the dataset with images from preoperative endometrial biopsy samples.

In the pathological diagnosis of EC, one of the most clinically significant challenges is to distinguish low‐grade endometrioid carcinoma from one of its high‐grade mimics, both exhibiting predominant adenoid structure morphology (Figure 5a). Another major challenge is to correctly determine histopathological subtypes from limited preoperative endometrial biopsy tissue. Two junior and two senior practicing pathologists manually interpreted 197 images showing adenoid structure morphology and 52 images collected from preoperative endometrial sampling, with subsequent comparison with predictions made by EC‐AI^HIS^. In the dataset with images showing adenoid structure morphology, the accuracies were 81% for the junior pathologists and 85% for the senior pathologists. EC‐AI^HIS^ achieved an accuracy of 88%, outperforming both groups of pathologists (Figure 5b–d). EC‐AI^HIS^ achieved an AUC of 0.909, and the detailed performance of EC‐AI^HIS^ is shown in Figure 5e,f. In the dataset with images from preoperative endometrial biopsy samples, the accuracies were 86% for the senior pathologists and 78% for the junior pathologists. EC‐AI^HIS^ achieved an accuracy of 77%, which was lower than that of the senior pathologists and comparable to that of the junior pathologists (Figure 6a,b). EC‐AI^HIS^ achieved an AUC of 0.838, and the detailed performance of EC‐AI^HIS^ is shown in Figure 6c–e.

### 3.4 Performance of Pathologists With EC‐AI^HIS^ Assistance

**FIGURE 7:**
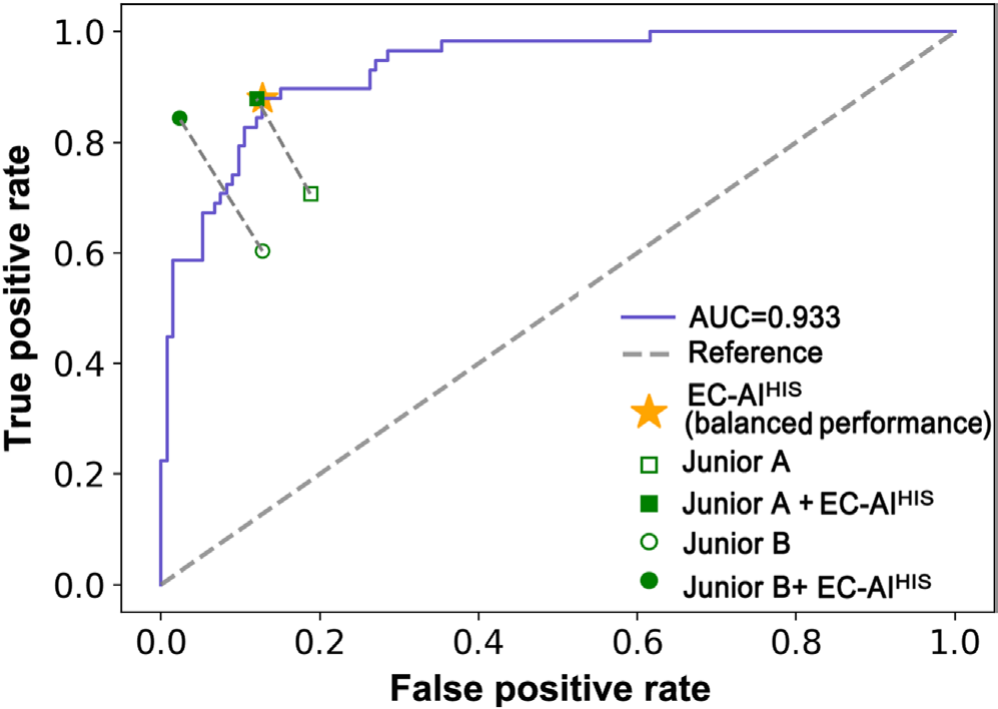
Performance of junior pathologists with EC‐AI^HIS^ assistance. The two junior pathologists’ original performances are denoted by unfilled dots, and those with EC‐AI^HIS^ assistance by filled dots. The star denotes the performance of EC‐AI^HIS^ in the ‘balanced performance’ setting.

To determine whether EC‐AI^HIS^ could be used to improve the performance of the junior pathologists, an additional 191 image cohort (not overlapping with the 197 and 52 images) was randomly selected from the testing sets. EC‐AI^HIS^‐predicted results were presented to the two junior pathologists for reference. The accuracy of the two junior pathologists, assisted by EC‐AI^HIS^, was markedly improved, increasing from 78.0% to 92.7% and from 79.6% to 94.8%, respectively. The sensitivity was improved the most, increasing from 70.7% to 87.9% and from 60.3% to 84.5%, respectively. Meanwhile, the specificity increased from 81.2% to 88.0% and from 87.2% to 97.7%, respectively (Figure 7).

### 3.5 Stratification Analysis of EC‐AI^HIS^ for Molecular Subtypes of EC

**FIGURE 8:**
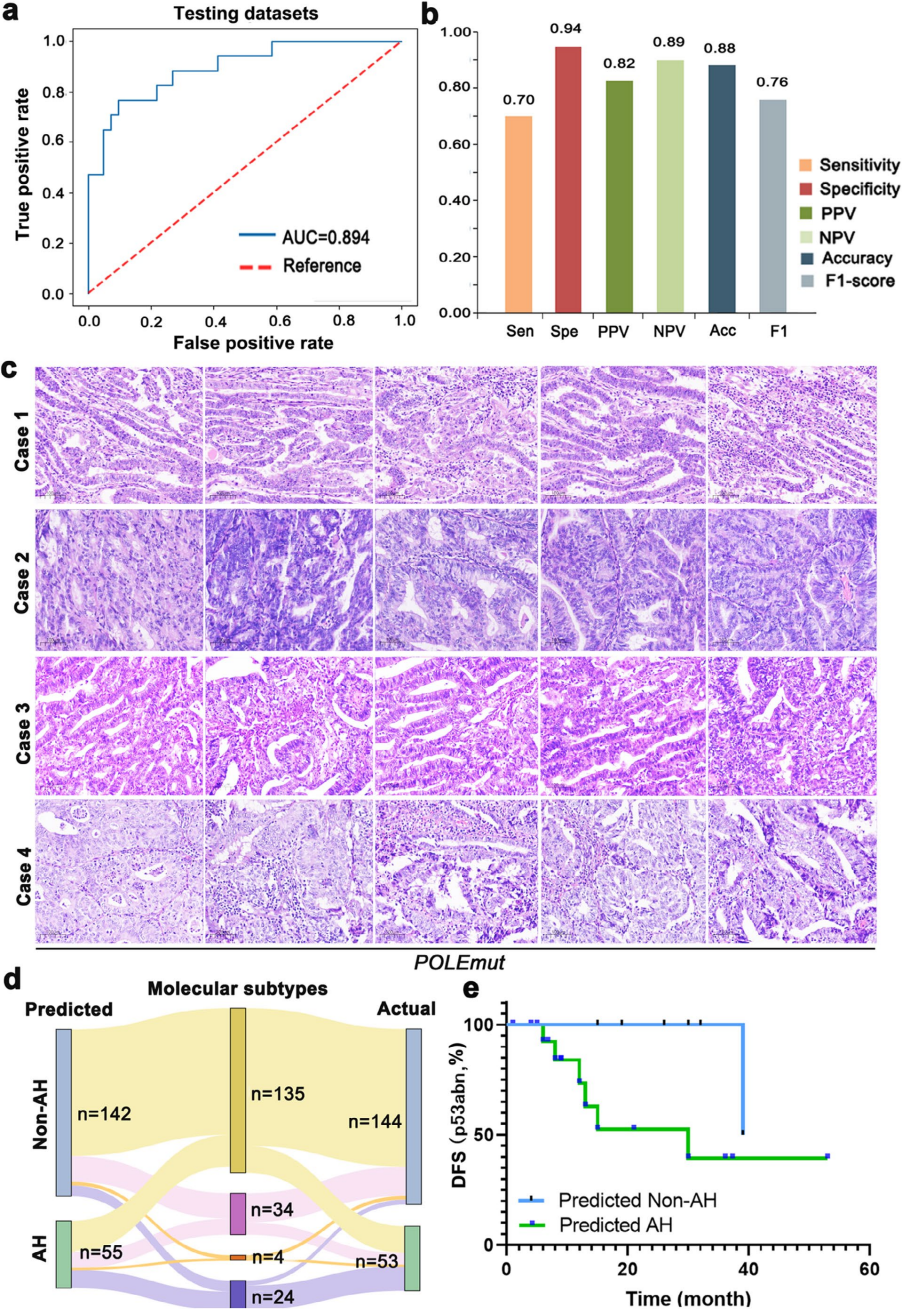
Association of EC‐AI^HIS^ and the molecular subtypes of endometrial carcinoma. (a) The AUC of EC‐AI^HIS^ in the molecular typing dataset. (b) Sensitivity, specificity, positive predictive value (PPV), negative predictive value (NPV), accuracy, and F1‐score of EC‐AI^HIS^ in the molecular typing dataset. (c) Representative histological images of the four cases with POLE mutation. (d) Alluvial plot showing distributions of nonaggressive and aggressive histological types in the four molecular typing subgroups. (e) Kaplan–Meier analysis of disease‐free survival (DFS) in the EC‐AI^HIS^‐predicted histotypes for patients of the p53abn subtype.

We further investigated the association between EC‐AI^HIS^ and the molecular subtypes of EC. We formed an independent dataset with 197 images from the internal dataset and classified these images into one of the four molecular subtypes, including POLE‐mutant (2.0%), mismatch repair‐deficient (MMR‐d) (17.3%), p53abn (12.1%), and no specific molecular profile (NSMP) (68.6%), based on information on POLE mutation and immunohistochemical information of MMRs (MLH1, PMS2, MSH2, MSH6) and P53. The AUC of EC‐AI^HIS^ for the molecular typing dataset was 0.894 (Figure 8a). Classification metrics, including sensitivity, specificity, PPV, NPV, accuracy, and F1‐score of EC‐AI^HIS^ are shown in Figure 8b. There were four cases of POLE‐mutant (Figure 8c), and one of them (case 2, certain regions exhibiting significant solid structure and notable inflammatory cell infiltration) was inaccurately classified as aggressive subtypes. The allocation of nonaggressive histological types and aggressive histological types in the four molecular subtype groups is shown in Figure 8d. The distribution of predicted histotypes from EC‐AI^HIS^ closely approximates the actual distribution within each molecular subtype group. Furthermore, we conducted a survival risk stratification analysis by integrating the four molecular subtypes with EC‐AI^HIS^. Notably, patients of the p53abn subtype were classified into different risk groups with different DFS outcomes (log‐rank *p* = 0.048, Figure 8e), indicating that EC‐AI^HIS^ may provide more comprehensive prognostic information. For the other three molecular subtypes, DFS did not show significant differences, as there were fewer cases of tumor progression log‐rank *p* > 0.99 (POLE‐mutant), *p* = 0.44 (MMR‐D), *p* = 0.50 (NSMP), (Figure S2).

### 3.6 Model Interpretability and Analysis of False Results

**FIGURE 9:**
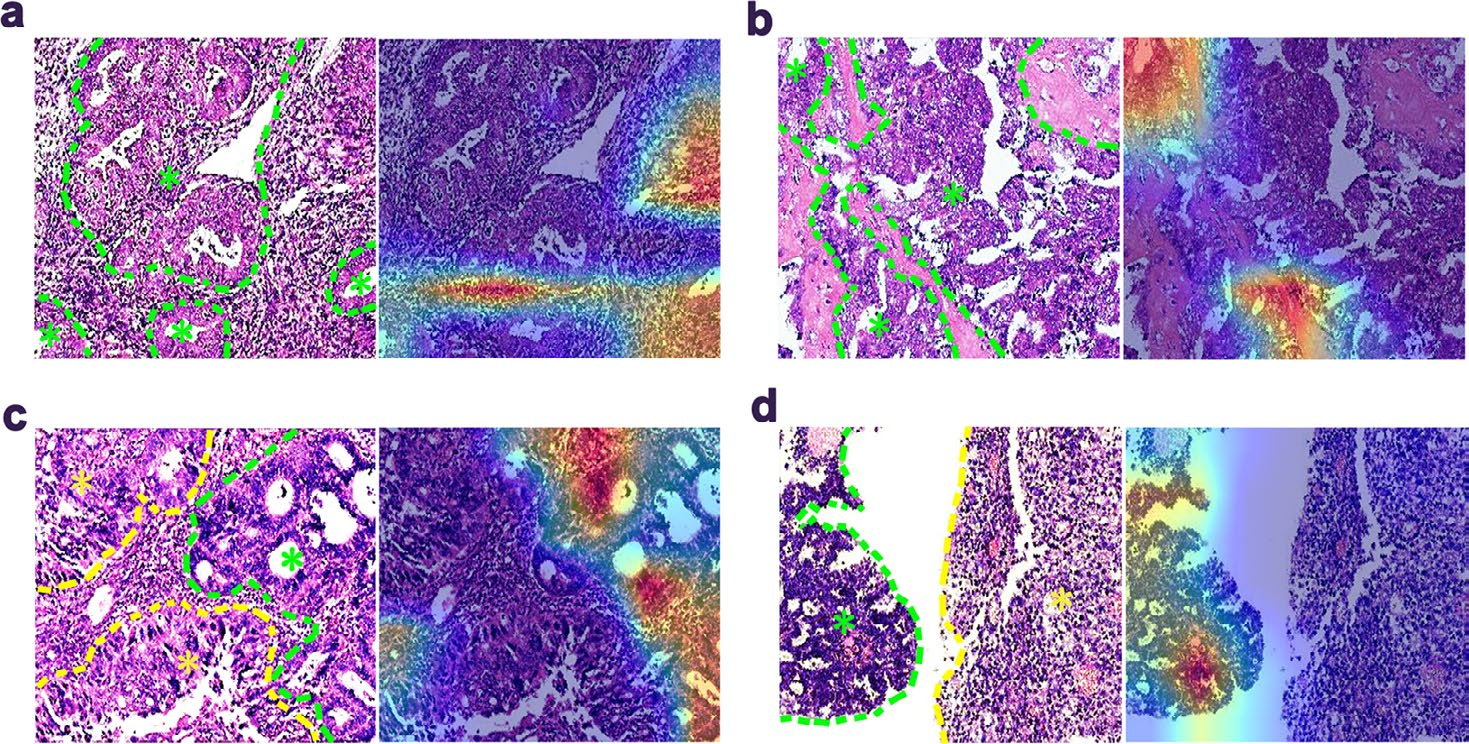
Visualization of morphological characteristics in H&E image patches from representative cases misclassified by EC‐AI^HIS^. (a) and (b) EC‐AI^HIS^ incorrectly focuses on nontumor mesenchymal components. Green dashed lines and asterisks indicate the tumor region. (c) and (d) EC‐AI^HIS^ focus on the nonaggressive regions within a case of endometrial mixed carcinoma (c). Green dashed lines and asterisks indicate the endometrioid carcinoma region, yellow dashed lines and asterisks indicate the serous carcinoma region, and a case of endometrial dedifferentiated carcinoma (d). Green dashed lines and asterisks indicate the endometrioid carcinoma region, and yellow dashed lines and asterisks indicate the undifferentiated carcinoma region.

In the attention heat maps, the red region is highly informative and highlights the histomorphological features relevant to the prediction outcomes of EC‐AI^HIS^ (Figure 9). The comprehensive accuracy percentage was 82.2%. The performance of the EC‐AI^HIS^ model on the number of cases with correct diagnoses in different datasets and different histotypes is presented in Tables S2 and S3. Manual visual inspection showed that the histomorphological features contributing most to the prediction made by EC‐AI^HIS^ include (1) densely packed nuclear region exhibiting a distinct solid lamellar structure; (2) complex papillary and conspicuously heterotypic glandular structure; (3) high‐grade cytologic features with marked nuclear pleomorphism, abnormal chromatin, macronucleoli, increased nucleus to cytoplasm ratio, and conspicuous mitotic activity with atypical mitoses. Besides, we also analyzed the false cases by visualization of the morphological characteristics extracted by EC‐AI^HIS^. Two primary reasons for misclassification of cases include (1) emphasis on the mesenchymal components of EC and (2) the identification of relatively nonaggressive regions within EC tissues containing components with varying degrees of differentiation. The H&E image patch in Figure 9 demonstrates several examples of misclassified cases by EC‐AI^HIS^.

## 4 Discussion

The 2023 FIGO staging system fully emphasizes the importance of the histological type of EC [28]. Aggressive histological types were categorized into worse stages than nonaggressive histological type [29, 30, 31]. In cases confined to the endometrium, nonaggressive histological types were classified as stage IA1, while aggressive histological types were categorized as stage IC. In cases with myometrial involvement and no/focal lymphovascular space involvement (LVSI), nonaggressive histological types with less than half of the myometrium invasion and half/more than half of the myometrium were categorized as stage IA2 or IB, respectively, while aggressive histological types were classified as IIC regardless of the depth of myometrial infiltration. Thus, accurate diagnosis of histological type directly guides clinical decision‐making and determines risk stratification.

Traditionally, pathological diagnosis relies heavily on the judgment of trained pathologists and sometimes requires the assistance of immunohistochemistry, fluorescence in situ hybridization, and sequencing. The process is subject to the experience of the pathologist and can be costly and time‐consuming. In addition, the dearth of pathologists has become a real bottleneck to efficient and accurate medical care, especially in underdeveloped countries and regions [32, 33]. The status quo is being changed with the advent of AI tools in pathology. DL‐assisted tools have shown excellent accuracy in pathology diagnosis in areas such as melanoma, breast, colorectal, and prostate cancer, underscoring its validity and utility in clinical application [34, 35, 36, 37]. The potential of DL to accurately identify EC and its histologic correlates of aggressive behavior from pathological images has been demonstrated in several studies (Table S4). Some reports showed promising results using a random forest algorithm or convolutional neural networks (CNN) to help classify benign, premalignant, and malignant endometrial tissues [38, 39, 40, 41, 42]. Moreover, successful DL models can also be used to predict recurrence, survival, and even molecular and genomic features. Several reports implemented the prediction of microsatellite status in EC cases using DL methods [43, 44, 45]. Hong and colleagues developed a customized multi‐resolution deep convolutional neural network to predict molecular subtypes of EC and 18 common gene mutations [19]. Fremond S. et al. implemented an interpretable DL model, which could accurately identify morpho‐molecular correlates, outperforming the molecular classification at predicting 5‐year recurrence‐free survival [20]. By using multiplexed immunofluorescence for the simultaneous visualization and quantification of CD68 ^+^ macrophages, CD8 ^+^ T cells, FOXP3 ^+^ regulatory T cells, PD‐L1/PD‐1 protein expression, and tumor cells from 250 patients, Daniel Jiménez‐Sánchez and colleagues trained a multilevel DL model to accurately assess the risk of recurrence in EC [22]. Using convolutional neural networks for extracting histologic features and a vision transformer, Goyal and colleagues constructed a model, EndoNet, to classify low‐grade EC (endometroid carcinoma grades 1 and 2) and high‐grade EC (endometroid carcinoma grade 3, uterine serous carcinoma, carcinosarcoma) [46]. Notably, a recent study by Wang et al. proposed TR‐MAMIL, a DL framework for classifying aggressive versus nonaggressive EC and predicting tumor mutational burden (TMB) from whole‐slide images using TCGA data [47]. While TR‐MAMIL achieved strong performance (AUC 0.88), it included a limited spectrum of aggressive subtypes (G3 endometrioid and serous carcinoma), with a cohort of 529 patients. In contrast, the current study developed EC‐AI^HIS^ based on a larger and more diverse multicenter cohort of 1464 patients, encompassing a broader range of aggressive histological types—including not only G3 endometrioid and serous carcinomas but also clear cell, mixed, undifferentiated, dedifferentiated, carcinosarcoma, and mesonephric‐like carcinomas—thus better reflecting real‐world pathological diversity. Using a fivefold cross‐validation strategy, our model achieved an AUC of 0.879 with an interval of 0.865–0.909. Prediction efficiency was robustly validated on external samples from another medical center, with AUCs of 0.911 and 0.859 in the internal and external cohorts, respectively. Furthermore, EC‐AI^HIS^ maintained strong performance on images obtained from a different scanner (AUC 0.925) and on poor‐quality slides (AUC 0.818), demonstrating high robustness and generalizability under varied clinical conditions. Critically, unlike previous models that predominantly utilized surgical resection specimens, EC‐AI^HIS^ was also tested on preoperative endometrial biopsy samples—a common but challenging clinical scenario—where it showed comparable performance to junior pathologists and significantly improved diagnostic accuracy when used as an assistive tool.

According to the 2023 FIGO staging criteria, FIGO grade 3 endometrioid carcinoma is regarded as an aggressive histologic type. Identification of the specific histological type of predominantly solid endometrial adenocarcinoma that confuses the pathologist does not affect the stage of the tumor. Due to the existence of many morphologic variations of endometrioid adenocarcinoma, including mucinous differentiation, squamous differentiation, morular metaplasia, cytoplasmic clearing, villo‐glandular variant, small nonvillous papillae, sex cord‐like pattern, and so on, the biggest diagnostic challenge for pathologists is to differentiate the FIGO grade 1/2 endometrioid carcinoma and one of its aggressive mimics, both showing adenoid structure morphology [48, 49, 50]. For each variant, a differential diagnosis with similar aggressive histological types is required. In addition, endometrial sampling is a fundamental step in preoperative workup. The histological type of EC is the most important factor that has to be assessed by preoperative endometrial sampling [51]. It aims to predict the three aforementioned factors, to determine whether neoadjuvant chemotherapy is required, to tailor the surgery approach, and the scope of surgery. The incorrect histological type evaluation of preoperative endometrial sampling may lead to a wrong therapeutic approach. Due to the limited tissue obtained, the heterogeneity of the tumor could not be reflected. Occasionally, the amount of tissue obtained is so minimal that a diagnosis cannot be reached, and further auxiliary tests, such as immunohistochemical staining or molecular detection, cannot be performed. Thus, determining the histological types of EC in preoperative endometrial sampling is another major challenge in clinical work. We then validated the performance of the model in cases presenting with adenoid structural morphology and in preoperative EC sampling. In the dataset showing adenoid structure morphology, EC‐AI^HIS^ showed comparable performance with the senior pathologists in terms of accuracy, demonstrating the feasibility of EC‐AI^HIS^ in discriminating between aggressive and nonaggressive EC. In contrast, the performance of EC‐AI^HIS^ in the dataset obtained from preoperative endometrial sampling was not as favorable as that observed in the postoperative samples. We postulated that this may be attributed to the presence of a higher proportion of non‐neoplastic components in the biopsy tissue samples, such as normal endometrial tissue, endometrial tissue with atypical hyperplasia, bleeding, mucus, and cellulose exudation, which are prone to causing interference. Moreover, it is worth noting that EC‐AI^HIS^ outperformed both groups of pathologists in sensitivity, suggesting that it may serve as a valuable tool to alert pathologists to pay more attention to certain cases and conduct careful reevaluation. And, as we expected, the performance of the junior pathologists could be markedly improved with the assistance of EC‐AI^HIS^, suggesting it has vast potential to be used in a man–machine collaboration pattern for classifying aggressive and nonaggressive EC, particularly in settings with limited pathological expertise.

Noteworthy, the 2023 FIGO staging system of EC not only reflects the innate histopathological but also the molecular nature of EC. According to the new staging system, the previous I and II stages were rearranged in patients with POLE‐mutant and p53abn, which displayed particularly distinct, favorable, and adverse outcomes in the early stage, respectively [52, 53]. Two previous studies have developed DL models for H&E‐based prediction of molecular EC classification [19, 20]. Both models achieved high accuracy, suggesting their potential for clinical application to help pathologists determine molecular subtypes and mutations of EC without performing IHC or sequencing. Among the current study cohort, 197 cases had definite molecular profiles of POLE‐mutant, MMR‐d, and p53abn. In this subset, EC‐AI^HIS^ achieved a 0.894 AUROC in classifying samples into aggressive or nonaggressive subtypes, with an F1 score of 0.76. However, due to the limitations of the samples, especially the small number of cases with molecular typing and POLE mutations, it is difficult to design a multi‐resolution DL architecture at this time. Further development of the model, including enlargement of the training dataset with molecularly classified EC, is needed to refine risk stratification and to guide treatment decisions.

In conclusion, this study highlights the potential of utilizing H&E histology image‐based DL to differentiate aggressive from nonaggressive histological types in EC. The developed model EC‐AI^HIS^ was validated through extensive analysis of two retrospective datasets and demonstrates remarkable predictive performance, robustness, and generalizability, indicating its vast potential to facilitate the diagnosis of EC by pathologists.

It is important to acknowledge several limitations of this study. First, the model was trained and validated on image patches rather than whole‐slide images, which may not fully capture the spatial heterogeneity of tumors. In addition, the number of cases for certain rare aggressive histological subtypes, such as undifferentiated carcinoma, dedifferentiated carcinoma, clear cell carcinoma, mixed carcinoma, and mesonephric‐like carcinoma, was relatively small. Future studies should incorporate more cases of these rare subtypes to improve the robustness and predictive accuracy of the model. Second, mimics of EC, including endometrial premalignant lesions and reactive atypia, were not included in the dataset. This limitation may restrict the algorithm's applicability in routine clinical practice, where such differential diagnoses are common. Third, although clinical factors such as age and menopause status differed significantly between aggressive and nonaggressive groups, their integration did not enhance the model's classification performance. This finding warrants further investigation, and more sophisticated modeling approaches that integrate multimodal data may help better account for clinical heterogeneity. Furthermore, future models should incorporate molecular features such as mismatch repair (MMR) status, which is increasingly relevant for treatment decisions in endometrial cancer. Finally, prospective validation is essential to confirm the clinical utility and generalizability of the model before its implementation in real‐world settings.

## Author Contributions

**Lingmei Li:** conceptualization; writing, original draft preparation. **Pengbo Wang:** data curation; formal analysis. **Wenjuan Ma** and **Xiaofeng Li:** investigation; software. **Changyu Geng:** methodology. **Dandan Chen**, **Ge Qiao**, **Jingyi Wang**, **Shi Zhang**, **Ningrui Feng**, **Xiaozhi Liu** and **Zhongmin Jiang:** project administration. **Lu Cao**, **Yanan Gao**, **Ming Liu**, **Yaomei Ma**, **Su Zhang** and **Huiting Xiao:** resources and validation. **Lisha Qi:** supervision; visualization; writing, review and editing.

## Funding

This work was supported by Tianjin Key Medical Discipline Construction Project (TJYXZDXK‐3‐016C), National Natural Science Foundation of China (81700103, 82002813, 82072004, 82202909, and 82272074), Tianjin Health Technology Project (TJWJ2023MS053), The Science & Technology Development Fund of Tianjin Education Commission for Higher Education (2021KJ193) and Tianjin Municipal Science and Technology Program (24ZYCGSY00650).

## Disclosure

Animal Studies: The authors have nothing to report.

## Ethics Statement

Approval of the research protocol by an Institutional Reviewer Board: This multicenter study has received ethical approval from the Ethics Committee and Institutional Review Boards of Tianjin Medical University Cancer Institute and Hospital and Tianjin Fifth Central Hospital.

## Consent

The authors have nothing to report.

## Conflicts of Interest

The authors declare no conflicts of interest.

## Supporting information


**Figure S1:** Feature importance ranking by SHAP values in diagnostic model based on the XGBoost algorithm. (a) Age and menopause are sorted according to the sum of the SHAP values of all patients, and SHAP values are used to represent the distribution of the influence of each feature on the output of the XGBoost model. (b) The standard bar chart is drawn and sorted using the average absolute value of each feature shape value in the XGBoost model. (c, d) The pathological images, class activation mapping, and waterfall chart of the XGBoost model predicted the histotype of EC in two cases, nonaggressive (c) and aggressive (d).

**Figure S2:** Kaplan–Meier analysis of disease‐free survival. The EC‐AIHIS‐predicted hisotypes for patients of the POLE‐mutant (a), MMR‐D (b), and NSMP (c) subtypes. MMR‐D: mismatch repair‐deficient; NSMP: no specific molecular profile.

**Table S1:** Clinical characteristics of endometrial carcinoma patients in training set.

**Table S2:** The diagnostic performance of EC‐AI^HIS^ in different datasets.

**Table S3:** The diagnostic performance of EC‐AI^HIS^ in both internal and external datasets across various histological types.

**Table S4:** Literature review of deep learning models for endometrial cancer based on histopathology images.

## Data Availability

The datasets generated and analyzed during the current study are not publicly available due to the reuse of the information repository of cases. Use of relevant case information must be approved by the ethics committee. Please email any request for academic use of the imaging data to either the corresponding author (lqi01@tmu.edu.cn) or the first author (lilingmei@tjmuch.com).
