# Supplementary figures and images for: Histopathology Images‐Based Deep Learning Prediction of Histological Types in Endometrial Cancer
Source: Cancer Med. 2025 Dec 30;15(1):e71509. doi: 10.1002/cam4.71509 (PMC12753328; doi:10.1002/cam4.71509)

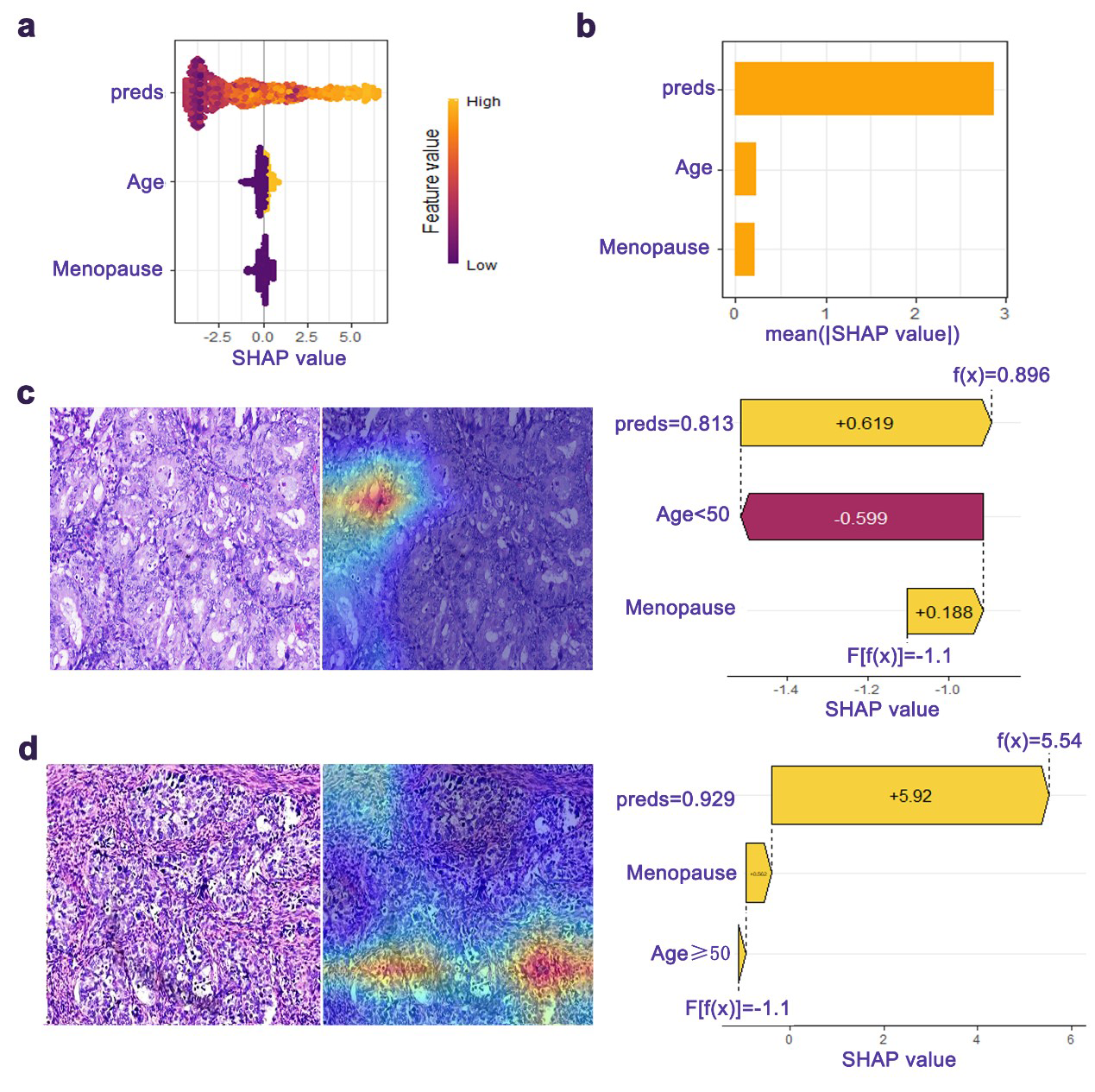

Supplement: Supplementary file 1 — Figure S1: Feature importance ranking by SHAP values in diagnostic model based on the XGBoost algorithm. (a) Age and menopause are sorted according to the sum of the SHAP values of all patients, and SHAP values are used to represent the distribution of the influence of each feature on the output of the XGBoost model. (b) The standard bar chart is drawn and sorted using the average absolute value of each feature shape value in the XGBoost model. (c, d) The pathological images, class activation mapping, and waterfall chart of the XGBoost model predicted the histotype of EC in two cases, nonaggressive (c) and aggressive (d). [file CAM4-15-e71509-s003.tif]

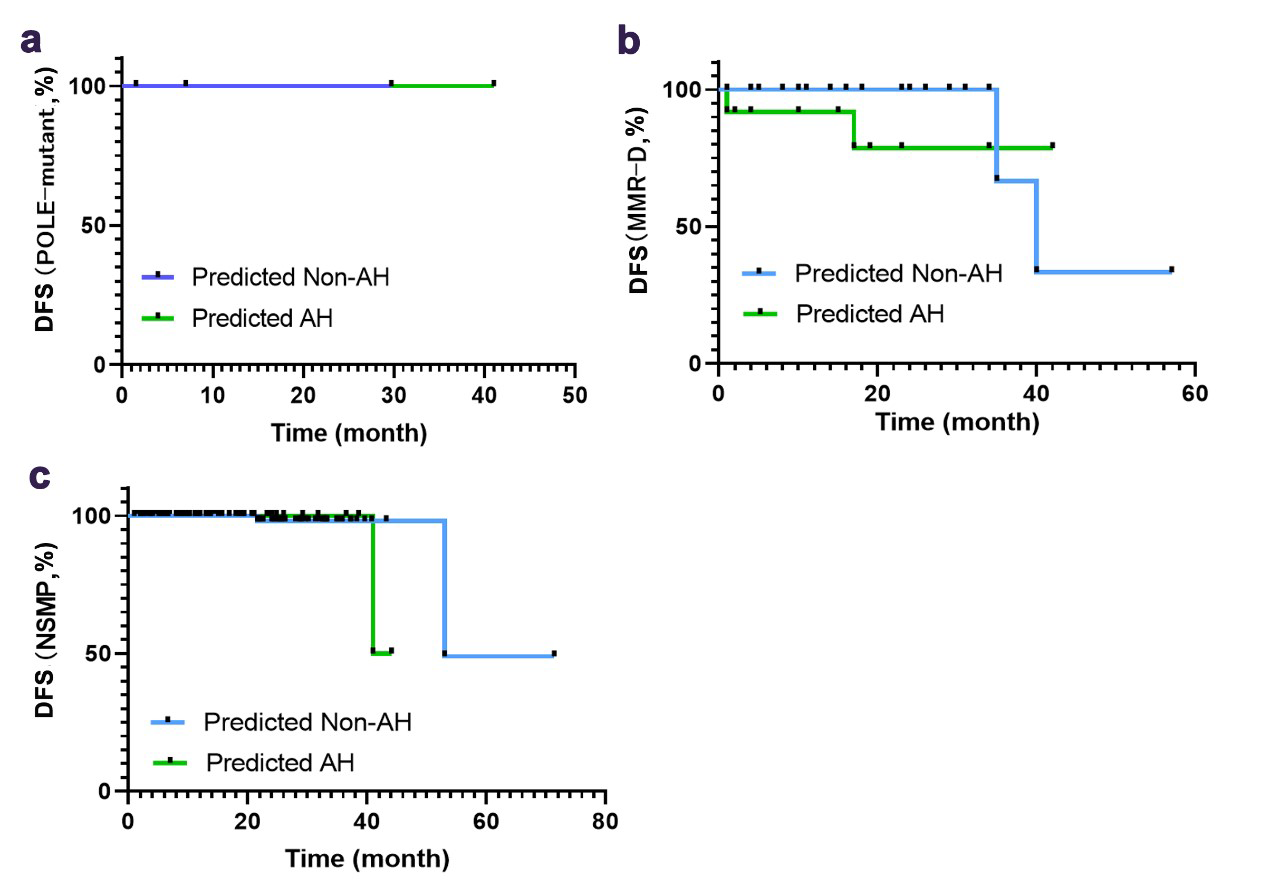

Supplement: Supplementary file 2 — Figure S2: Kaplan–Meier analysis of disease‐free survival. The EC‐AIHIS‐predicted hisotypes for patients of the POLE‐mutant (a), MMR‐D (b), and NSMP (c) subtypes. MMR‐D: mismatch repair‐deficient; NSMP: no specific molecular profile. [file CAM4-15-e71509-s001.tif]
