# Supplementary material for: Histopathology Images‐Based Deep Learning Prediction of Histological Types in Endometrial Cancer
Source: Cancer Med. 2025 Dec 30;15(1):e71509. doi: 10.1002/cam4.71509 (PMC12753328; doi:10.1002/cam4.71509)
Supplement: Supplementary file 3 — Table S1: Clinical characteristics of endometrial carcinoma patients in training set. [file CAM4-15-e71509-s004.docx]

| **Table S1. Clinical characteristics of endometrial carcinoma patients in training set.** | | | | | |  |
| --- | --- | --- | --- | --- | --- | --- |
| Characteristics | All (n=746) | Non-aggressive histological type (n=523) | Aggressive histological type (n=223) | *p-Value* | | |
| Age, mean(range) | ＜0.05* | | | | |  |
| ≤50 | 205(27.5) | 163(31.2) | 42(18.8) | |  |  |
| ＞50 | 541(72.5) | 360(68.8) | 181(81.2) | |  |  |
| Vaginal bleeding |  |  |  | | 0.138 |  |
| No | 115(15.4) | 75(14.3) | 40(17.9) | |  |  |
| Yes | 631(84.6) | 448(85.7) | 183(82.1) | |  |  |
| Menarche age |  |  |  | | 0.067 |  |
| ≤14 | 272(49.9) | 260(49.7) | 112(50.2) | |  |  |
| ＞14 | 374(50.1) | 263(50.3) | 111(49.8) | |  |  |
| Menopause status |  |  |  | | 0.05* |  |
| No | 262(35.1) | 205(39.2) | 57(25.6) | |  |  |
| Yes | 484(64.9) | 318(60.8) | 166(74.4) | |  |  |
| Childbearing history |  |  |  | | 0.073 |  |
| No | 37(5.0) | 28(5.4) | 9(4.0) | |  |  |
| Yes | 709(95.0) | 495(94.6) | 214(96.0) | |  |  |
| Abortion |  |  |  | |  |  |
| No | 301(40.3) | 206(39.4) | 95(42.6) | | 0.413 |  |
| Yes | 445(59.7) | 317(60.6) | 128(57.4) | |  |  |
| Family history |  |  |  | | 0.853 |  |
| No | 562(75.3) | 395(75.5) | 167(74.9) | |  |  |
| Yes | 184(24.7) | 128(24.5) | 56(25.1) | |  |  |

*indicates statistically significant.
