# Supplementary material for: Histopathology Images‐Based Deep Learning Prediction of Histological Types in Endometrial Cancer
Source: Cancer Med. 2025 Dec 30;15(1):e71509. doi: 10.1002/cam4.71509 (PMC12753328; doi:10.1002/cam4.71509)
Supplement: Supplementary file 4 — Table S2: The diagnostic performance of EC‐AIHIS in different datasets. [file CAM4-15-e71509-s006.docx]

**Table S2. The diagnostic performance of EC-AI^HIS^ in different datasets**

| Category | Internal datasets | External datasets | Different scanner datasets | Poor-quality datasets | Adenoid structure datasets | Preoperative sampling endometrial datasets | Molecular subtypes datasets |
| --- | --- | --- | --- | --- | --- | --- | --- |
| The accuracy  (number of correct predictions/ number of patients) | 82.0%  (875/1067) | 79.4%  (54/68) | 87.5%  (56/64) | 76.7%  (66/86) | 75.4% (485/643) | 78.8%  (41/52) | 87.4%  (173/198) |
