# Supplementary material for: Histopathology Images‐Based Deep Learning Prediction of Histological Types in Endometrial Cancer
Source: Cancer Med. 2025 Dec 30;15(1):e71509. doi: 10.1002/cam4.71509 (PMC12753328; doi:10.1002/cam4.71509)
Supplement: Supplementary file 5 — Table S3: The diagnostic performance of EC‐AIHIS in both internal and external datasets across various histological types. [file CAM4-15-e71509-s002.docx]

**Table S3. The diagnostic performance of EC-AI^HIS^ in both internal and external datasets across various histological types**

| Histotypes | The accuracy on the internal datasets (number of correct predictions/number of patients) | The accuracy on the external datasets (number of correct predictions/number of patients) |
| --- | --- | --- |
| EEC Grade 1-2 | 81.6% (612/750) | 82.7% (24/29) |
| EEC Grade 3 | 78.1%(118/151) | 42.8% (3/7) |
| ESC | 85.0%(91/107) | 62.5% (15/24) |
| ECCC | 91.3% (21/23) | 100% (1/1) |
| EMixC | 100%(18/18) | 85.7% (6/7) |
| ECS | 72.7%(8/11) | 0.0%(0/0) |
| EUC | 100%(4/4) | 0.0%(0/0) |
| EDC | 100%(2/2) | 0.0%(0/0) |
| MLA | 100%(1/1) | 0.0%(0/0) |

EEC: Endometrial endometrioid carcinoma; ESC: endometrial serous carcinoma; ECCC: endometrial clear cell carcinoma: EMixC: endometrial mixed carcinoma; ECS: endometrial carcinosarcoma; EUC: endometrial undifferentiated carcinoma; EDC: endometrial dedifferentiated carcinoma; MLA: mesonephric-like adenocarcinoma.
