# Supplementary material for: Histopathology Images‐Based Deep Learning Prediction of Histological Types in Endometrial Cancer
Source: Cancer Med. 2025 Dec 30;15(1):e71509. doi: 10.1002/cam4.71509 (PMC12753328; doi:10.1002/cam4.71509)
Supplement: Supplementary file 6 — Table S4: Literature review of deep learning models for endometrial cancer based on histopathology images. [file CAM4-15-e71509-s005.docx]

**Table S4. Literature review of deep learning models for endometrial cancer based on histopathology images**

| Work | Model | Sample (N) | Included histological types | Prediction performance | Interpretability |
| --- | --- | --- | --- | --- | --- |
| Liu et.al  (2025)^45^ | MMR Net | 2079 | Not mentioned | Prediction of MMR-deficient EC with 0.895 AUC | Attention zero |
| Wang et.al  (2024)^47^ | TR-MAMIL | 529 | EEC; ESC | Classification of aggressive and non-aggressive EC with 0.89 AUC | Heat map |
| Goyal et.al  (2024)^46^ | EndoNet | 615 | EEC\|; ESC; ECS | Classification of low-grade and high-grade EC with 0.86-0.95 AUC | Attention map |
| Wang et.al  (2024)^44^ | DL | 529 | EEC | Assess MSI status of EC with 0.84-0.94 AUC | Attention map |
| [Fremond](https://pubmed.ncbi.nlm.nih.gov/?sort=pubdate&term=Fremond+S&cauthor_id=36496303) et.al  (2023)^20^ | im4MEC | 2028 | Not mentioned | Prediction of the four molecular classes in EC with 0.874 AUC | Attention map |
| [Zhang](https://pubmed.ncbi.nlm.nih.gov/?sort=pubdate&term=Zhang+Y&cauthor_id=37150803) et.al  (2023)^43^ | DL | 95 | Not mentioned | Prediction of microsatellite status in EC with 0.799 AUC | Heat map |
| Zhang et.al  (2022)^42^ | DL | 1190 | EEC; proliferative lesions | Classification of EC and non-EC with 0.928 AUC | Heat map |
| Hong et.al  (2021)^19^ | Panoptes | 456 | EEC; ESC | Classification into EEC or ESC histological subtypes with 0.969 AUC | Attention map |
| Zhang et.al (2021)^23^ | VGGNet-16 | 454 | EEC; proliferative lesions | Classification of endometrial lesions with 0.916-0.981 AUC | No |
| Papke Jr et.al (2021)^40^ | DL | 91 | Proliferative lesions; EIN; | Classification of endometrial tissue fragments as neoplastic-EIN or background-NL | Attention zero |
| Sun et.al  (2020)^41^ | HIENet | 500 | EEC; proliferative lesions | Classification for benign and malignant lesions with 0.9829 AUC | Heat map |
| [Makris](https://pubmed.ncbi.nlm.nih.gov/?sort=pubdate&term=Makris+GM&cauthor_id=28160459) et.al  (2020)^38^ | ANN-MPL | 416 | EEC; proliferative lesions | Classification for benign and malignant endometrial nuclei and lesions | No |
| [Downing](https://pubmed.ncbi.nlm.nih.gov/?sort=pubdate&term=Downing+MJ&cauthor_id=31157686) et.al  (2017)^39^ | DL | 148 | NL; EIN; EMCA | Classification for cases with error rates of 0-0.058 | Attention zero |

EC: Endometrial cancer; EEC: Endometrial endometrioid carcinoma; ESC: endometrial serous carcinoma; AUC: area under the curve; ECS: endometrial carcinosarcoma; DL: deep learning; NL: normal lesions, EIN: endometrial intraepithelial neoplasia; EMCA: endometrial malignant carcinoma; MMR: mismatch repair; MSI: microsatellite instability.
